# Supplementary material for: Association between Circulating MicroRNAs (miR-21-5p, miR-20a-5p, miR-29b-3p, miR-126-3p and miR-101-3p) and Chronic Allograft Dysfunction in Renal Transplant Recipients
Source: Int J Mol Sci. 2022 Oct 14;23(20):12253. doi: 10.3390/ijms232012253 (PMC9603156; doi:10.3390/ijms232012253)
Supplement: Supplementary file 1 [file ijms-23-12253-s001.zip › ijms-1936704-supplementary.pdf]

International Journal of *Molecular Sciences*

MDPI

Supplement:

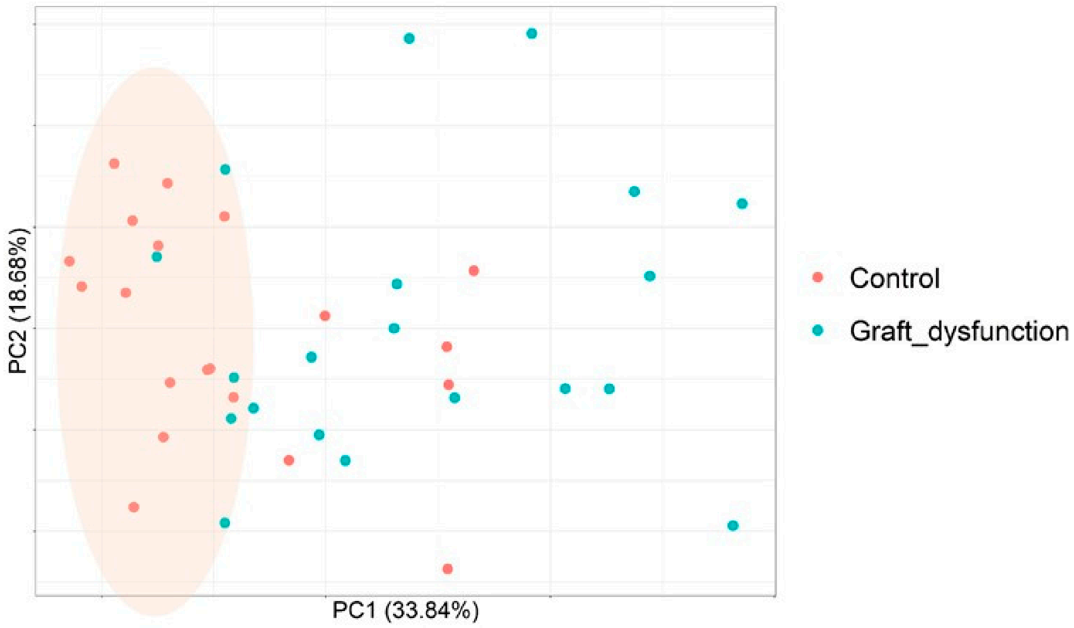

Figure S1. PCA plot

Principal Component Analysis (PCA) of differentially expressed miRNAs. PC1 explaining 33.84% variation and separated s while PC2 explained 18.6% of the variation. A scatter plot of PC1 and PC2 separated most samples of control group from graft dysfunction group.

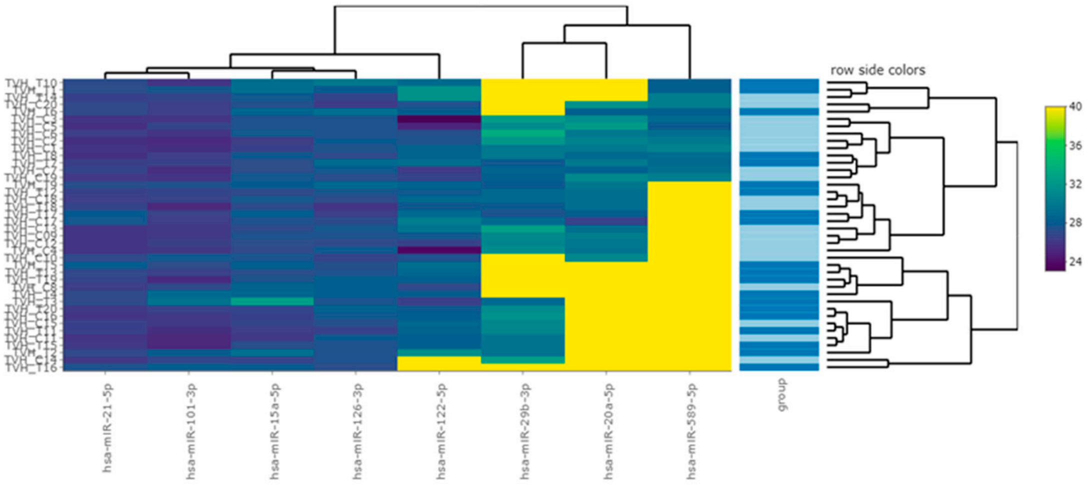

Figure S2. Clustering Analysis

Clustering was performed to visualize the correlations among the replicates and varying sample conditions. Up- and down-regulated microRNAs are represented in blue and yellow colors, respectively. The control and CAD group are represented in dark blue and light blue respectively. The 8 differential expressed microRNAs are selected for clustering analysis.

Table S1. Top 10 Enrichment GO terms of molecular function (MF)

GO term	Gene Ratio	Bg Ratio	P value
chromatin binding	49/383	191/2687	1.09E-05
extracellular matrix structural constituent	17/383	46/2687	9.64E-05
protein domain specific binding	58/383	260/2687	0.000146592
kinase binding	65/383	310/2687	0.000387717
copper ion binding	8/383	18/2687	0.001872214
transcription regulatory region sequence-specific DNA binding	76/383	404/2687	0.003553698
S-adenosylmethionine-dependent methyltransferase activity	9/383	24/2687	0.003979518
DNA-binding transcription activator activity	39/383	188/2687	0.00749833
2-oxoglutarate-dependent dioxygenase activity	7/383	18/2687	0.008728469
transmembrane receptor protein kinase activity	14/383	178/2689	0.013595668

*The top 10 terms GO term in the category of molecular function listed according to P-value.

Table S2. Top 10 Enrichment GO terms of cellular component (CC)

GO term	Gene Ratio	Bg Ratio	P value
endoplasmic reticulum lumen	28/388	80/2703	2.14E-06
platelet alpha granule lumen	14/388	28/2703	7.40E-06
collagen-containing extracellular matrix	35/388	125/2703	3.60E-05
cell surface	56/388	252/2703	0.000263661
collagen trimer	8/388	18/2703	0.001962232
protein kinase complex	14/388	51/2703	0.010179095
glial cell projection	6/388	16/2703	0.018895197
outer membrane	14/388	57/2703	0.026899253
integrin complex	4/388	11/2703	0.060192471
ruffle membrane	8/388	32/2703	0.076961095

*The top 10 terms GO term in the category of cellular function listed according to P-value
